# Incidence rates of dysvascular lower extremity amputation changes in Northern Netherlands: A comparison of three cohorts of 1991-1992, 2003-2004 and 2012-2013

**DOI:** 10.1371/journal.pone.0204623

**Published:** 2018-09-24

**Authors:** Behrouz Fard, Pieter U. Dijkstra, Roy E. Stewart, Jan H. B. Geertzen

**Affiliations:** 1 Department of Rehabilitation Medicine, University Medical Center Groningen, University of Groningen, Groningen, the Netherlands; 2 Roessingh Center for Rehabilitation, Enschede, the Netherlands; 3 Department of Oral and Maxillofacial Surgery, University Medical Center Groningen, University of Groningen, Groningen, the Netherlands; 4 Department of Public Health, University Medical Center Groningen, University of Groningen, Groningen, the Netherlands; Weill Cornell Medicine-Qatar, QATAR

## Abstract

**Objective:**

To analyze the incidence rates of dysvascular major lower extremity amputations (LEA) in Northern Netherlands in 2012–2013 compared to previous cohorts in 1991–1992 and 2003–2004.

**Design:**

Retrospective cohort study.

**Participants:**

Adults (N = 343) with first ever dysvascular LEA at ankle disarticulation or more proximal levels. The median age (interquartile range) was 74.2 years (64.5–81.9), 64% were male and 55% had been diagnosed with diabetes mellitus (DM).

**Main outcome measures:**

Crude and age-standardized incidence rates of major LEA per 100.000 person-years.

**Results:**

Crude incidence rate (IR) of first ever major LEA in 2012–2013 was 9.9 per 100.000 person-years, while the age-standardized IR was 7.7 per 100.000 person-years. A Poisson regression analysis showed that amputation rates among men were 2.3 times higher compared to women (95%CI 1.9–2.6), while in 2012–2013 the population aged >63 years had decreased amputation rates compared to 1991–1992. In the DM population the crude IR decreased from 142.6 per 100.000 person-years in 2003–2004 to 89.2 per 100.000 person-years in 2012–2013 (p<0.001).

**Conclusions:**

In 2012–2013 a decrease in age-standardized IR for the general population and a decrease in crude IR for the DM population were observed compared to cohorts from the previous two decades, despite considerable shifts in the age distribution of the Dutch population towards more elderly people and increased prevalence of DM. These findings might suggest that improved treatment of patients at risk of dysvascular amputations is associated with reduced incidence rates of major LEA at the population level.

## Introduction

In the past four decades over 90% of major lower extremity amputations (LEA) in the United States and Western European countries were related to peripheral arterial disease (PAD) and diabetes mellitus (DM)[1,2]. An estimated 6% percent of LEA was due to trauma, with the remaining proportion being related to a collection of causes such as cancer and congenital deficiencies[1]. Incidence rates for major LEA show considerable regional variation, as illustrated by a systematic review in 2011[3] in which the incidences were reported to range 3.6 to 69.4 per 100.000 person-years[3]. This variation reflects influence of regional factors such as the quality of health care services and demographic characteristics. However, differences in research methodology (e.g., definition of amputation levels) may also contribute to the variation of the reported incidences[4]. Recent studies report decreasing incidence rates of major LEA in Western European countries such as Germany[5], Spain[6], the United Kingdom[7], and Italy[8], while unchanged rates are reported in Hungary[9] and Ireland[10].

About 42 to 56% of major LEA occur among patients with DM[7,8,11], with the relative risk of major LEA for diabetic and non-diabetic populations ranging from 5.1 to 31.5[4,12,13]. In the past four decades extensive efforts have been made to improve the treatment of DM in regards to glycemic control and prevention of different types of complications[14]. For podiatric care in particular, prevention and treatment of foot ulcers have improved for DM patients[12,15]. Also, considerable advances have been made in the management of PAD: routine use of vascular ultrasonography, improved techniques for percutaneous angioplasty and extensive arterial bypass grafting[16–18].

Life expectancy in Western European countries has increased consistently[19], contributing to a relatively more elderly population and, subsequently, more years of exposure to DM and PAD. Considering these changes, an updated report of the regional incidence rates of major LEA in the Netherlands is expected to provide useful information for health care professionals and to facilitate international comparison. Although a previous study[20] reported unchanged incidence rates between 1991–1992 and 2003–2004 in the Northern region of the Netherlands, we hypothesize that changes in the age distribution in the regional population should be taken into account in analyzing the long term changes of incidence rates. To our knowledge this study is unique, in that major LEA incidences for a region were analyzed in detail, using data at the patient level, for a time frame spanning more than two decades. The aim of the study was to report the incidence rate of first ever dysvascular major LEA in 2012–2013 compared to rates of previous cohorts in 1991–1992[21] and 2003–2004[20]. We expected to find a modest decrease in amputation rates, in line with the reported trends in the literature.

## Methods

Data from cohorts 1991–1992, 2003–2004, and 2012–2013 were analyzed, where the cohorts 1991–1992 and 2003–2004 were available to our department. Twelve hospitals -comprising one academic hospital and eleven hospitals with operational endocrinology and surgery departments- in the Northern three provinces of the Netherlands (i.e., Groningen, Friesland and Drenthe) participated in the current study and the previous cohorts. Due to an absence of a nationwide medical registry in the Netherlands, medical records of amputation patients were accessed directly. The University Medical Ethical Committee was consulted and approval was obtained prior to data collection (M15.176087). Additionally, approval in each of the participating hospitals was provided by the Board of Directors or the local Medical Ethical Committees.

### Definitions and participants

International Organization for Standardization (ISO)[22] descriptors were used for the terminology of type and level of amputations. Major LEA was defined as any amputation at ankle disarticulation or more proximal level. All major LEA performed from January 1, 2012 through December 31, 2013 were included. Data collection at the hospitals was performed from January 1, 2015 through April 1, 2017. Dysvascular amputation was defined as any major LEA among patients with a recorded medical history of either or both DM and PAD at the time of or prior to the first major LEA according to hospital records or archived documents of the general practitioner. Additional details pertaining to the search strategy and inclusion are provided in the supplemental information S1 Table. For all three cohorts, patients who had undergone major LEA before the study period were excluded. For patients who had undergone multiple amputations during the study periods in the same limb (i.e., reamputation), the most proximal level of amputation was counted once. Patients who had both limbs amputated were counted once as a bilateral amputation. These measures were applied in order to avoid overestimating the incidence rate at the patient level (i.e., by adding up multiple amputations performed on the same patient) and double counting of patients in multiple cohorts. Patients with major LEA due to trauma, cancer, Complex Regional Pain Syndrome type-I, complication of orthopedic surgery, fulminant infection in otherwise healthy adults and congenital deficiencies were excluded. Population data of the adherence area of the hospitals involved were obtained through the open source database of the Dutch Central Bureau for Statistics (CBS)[23] from January 1, 1991 through January 1, 2013, with subpopulations specified by province of residence, sex and 5-years age groups.

### Statistical procedures

Due to changes of age distribution in the population between 1991 and 2013, both crude incidence rates (IR) and age-standardized IR were calculated per 100.000 person-years. The crude IR was calculated for the overall population and additionally by sex and age categories, using the corresponding subpopulation data for the denominators. The overall age-standardized IR and by sex were calculated following the direct method[24,25] using the 1991–1992 regional subpopulations as reference. The age distribution of the study population was observed to be left skewed, therefore median ages were presented. Accordingly, differences in median age by sex and DM status were analyzed using the Mann-Whitney U test and the trend of changing median age between 1991 and 2013 were analyzed using the Jonkheere-Terpstra test[26]. For calculating 95% confidence intervals (95%CI) for crude IR and age-standardized IR, a Poisson distribution was assumed. For the main analysis of changes in amputation rates in the general population, a Poisson regression analysis was performed with the three cohorts, sex and age as predictor variables and the corresponding subpopulations as the off-set variable. Interaction terms between predictor variables were explored. Diabetes status was unavailable for the 1991–1992 cohort, because of which we were unable to analyze changes of LEA incidence in the DM population through a Poisson regression. Incidence rates for the DM population could be only calculated for the 2003–2004 and 2012–2013 cohorts. Crude IR among DM patients and rate ratio’s (RR) were calculated, in which the DM population was estimated through DM prevalence rates by sex and age groups[23]. Changes in crude IR for the DM populations were analyzed using χ^2^ tests and by calculating 95% CI for the estimates. For all analyses statistical significance was set at α = 0.05. Microsoft Excel 2010, IBM SPSS Statistics 20 and OpenEpi 3.01 were used for the analyses.

## Results

In 2012 and 2013 a total of 382 patients underwent dysvascular major LEA, 39 patients were excluded due to major LEA prior to the study period. In 2012–2013 64% of the amputation patients were male and 55% were diagnosed with DM (Table 1). The median age of patients at the time of first major LEA between 1991–1992 and 2012–2013 decreased by 2.3 years (p = 0.019). In the 2012–2013 cohort the median age of men was 4.2 years lower than that of women (p = 0.028) and the median age of DM patients was 2.4 years lower than that of non-DM patients (p = 0.015).

**Table 1:** Characteristics of patients undergoing first major LEA in the three historic cohorts.

	1991–1992 n = 285	2003–2004 n = 299	2012–2013 n = 343	P value†
*Level of amputation*				
Transtibial	137 (48)	146 (49)	151 (44)	
Knee disarticulation	27 (10)	27 (9)	20 (6)	
Transfemoral	103 (36)	100 (34)	147 (43)	
Bilateral	18 (6)	25 (9)	25 (7)	
Male	168 (59)	180 (60)	220 (64)	
Diabetes*	-	150 (50)	190 (55)	
*Age at amputation*, *y*				
All	76.5 (68.6–82.7)	75.5 (66.5–82.6)	74.2 (64.5–81.9)	0.019
Men	73.8 (66.9–80.8)	72.0 (65.0–80.4)	72.7 (64.3–80.0)‡	0.248
Women	78.9 (72.7–84.2)	79.9 (72.0–84.5)	76.9 (66.2–84.2)‡	0.079
DM	-	75.5 (66.4–81.0)	72.8 (62.5–80.4)§	
Non-DM	-	75.7 (67.2–84.1)	75.2 (67.5–83.9)§	

NOTE. Values are numbers (%), except for age for which median (interquartile range) is provided.

* Diabetes status not available for the 1991–1992 cohort.

† Jonkheere-Terpstra test difference in median age 1991–1992 through 2012–2013.

‡ Mann-Whitney U test difference in median age of men vs women in 2012–2013, p = 0.028.

§ Mann-Whitney U test difference in median age of DM vs non-DM patients in 2012–2013, p = 0.015.

### Major LEA in the general population

**Fig 1:**
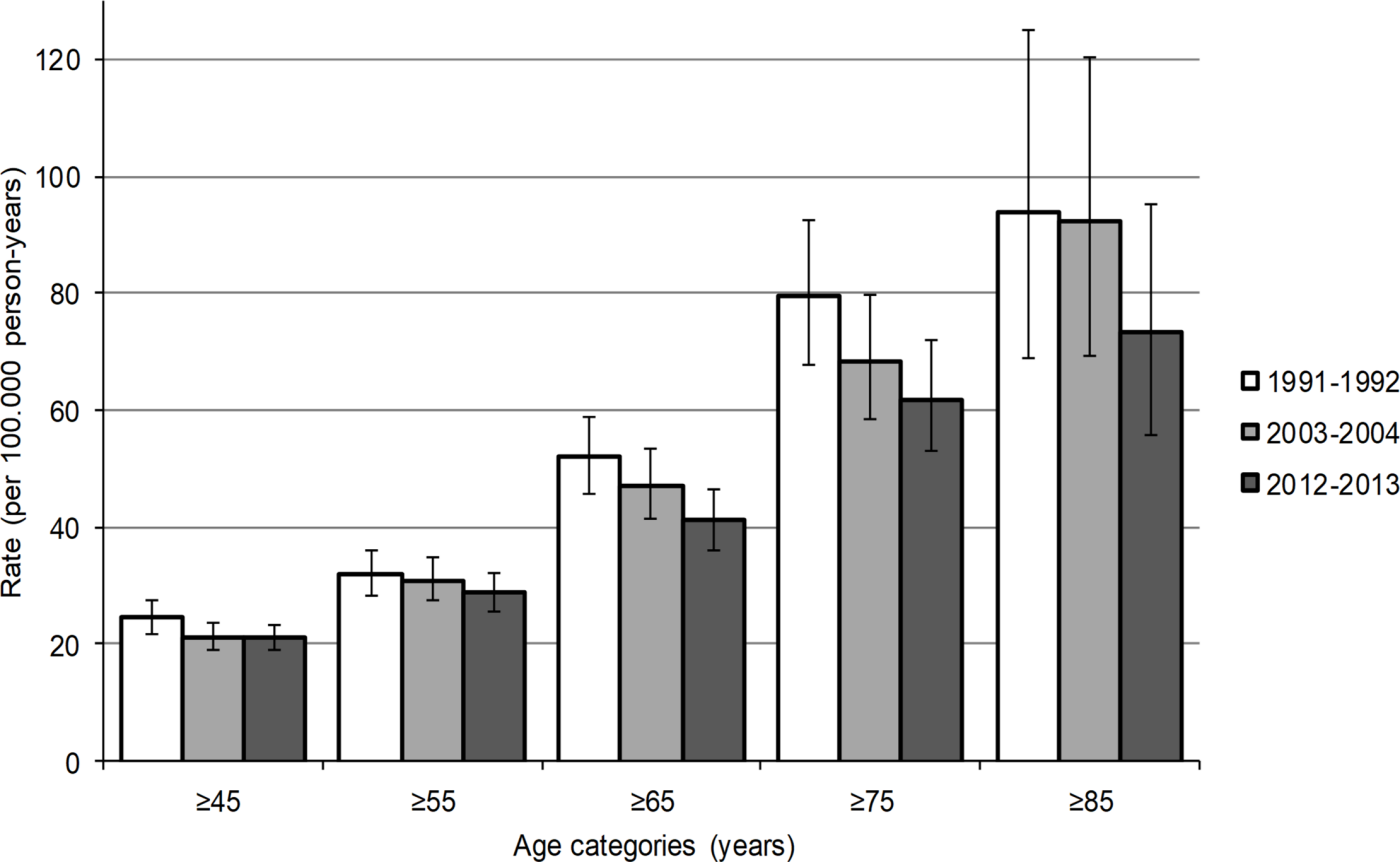
Crude incidence rate of major LEA for the three historic cohorts, by age categories. Error bars are 95% CI.

Crude IR of major LEA in 2012–2013 was 9.9 per 100.000 person-years, whereas in 1991–1992 and 2003–2004, crude IR was 8.9 and 8.8 per 100.000 person-years respectively (Table 2). Fig 1 is presented in order to illustrate the effect of increased age on incidence rates, as in all three cohorts higher crude IR were observed for the populations aged ≥45, ≥55, ≥65 and ≥75 years. With the 1991–1992 population as reference, the age-standardized IR of major LEA in 2012–2013 was 7.7 per 100.000 person-years overall, 9.4 per 100.000 person-years among men and 5.7 per 100.000 person-years among women (Table 2). The Poisson regression analysis (Table 3) provided an adequate model fit with no clear over or under dispersion. The amputation rates among men were 2.3 times (95%CI 1.9–2.6) higher compared to women, there were no interaction effects for sex between the cohorts and ages. A significant interaction effect was found between age and the three cohorts (p<0.001). Compared to 2012–2013 in 1991–1992 each one year increase in age was associated with 1.5% increase in amputation rate (RR 1.015; 95%CI 1.004–1.026) and in 2003–2004 each one year increase in age was associated with 1.3% increase amputation rate (RR 1.013; 95%CI 1.002–1.023). Applying the Poisson regression equation in S2 Table provides us with RR between the cohorts for a certain age. For example, applied to the data as presented in Table 3: in 2012–2013 for a 75 year old person the RR of major LEA was 0.84 compared to 1991–1992. Whereas, for a 45 year old person in 2012–2013 the RR was 1.31 compared to 1991–1992. For 2012–2013 the tilting point of RR deviating from 1.0 when compared to 1991–1992 was at 63 years and when compared to 2003–2004 at 70 years.

**Table 2:** Crude and age-standardized incidence rates of first ever major LEA for the general population.

	Crude IR (95% CI)			Age-standardized IR (95% CI)*	
	1991–1992	2003–2004	2012–2013	2003–2004	2012–2013
*Men and women*					
All ages	8.9 (7.9–9.9)	8.8 (7.9–9.9)	9.9 (8.9–11.1)	8.0 (6.9–9.1)	7.7 (6.8–8.7)
Age ≥45	24.6 (21.8–27.6)	21.3 (19.0–23.8)	21.1 (18.9–23.4)		
*Men*					
All ages	10.6 (9.1–12.3)	10.6 (9.1–12.2)	12.9(11.2–14.6)	11.4 (9.9–12.9)	9.4 (8.3–10.5)
Age ≥45	31.2 (26.7–36.3)	26.7 (22.9–30.8)	27.8 (24.3–31.8)		
*Women*					
All ages	7.3 (6.0–8.7)	7.1 (5.9–8.4)	7.1 (5.9–8.5)	6.5 (5.5–7.5)	5.7 (4.9–6.4)
Age ≥45	18.8 (15.6–22.5)	16.4 (13.6–19.5)	14.8 (12.3–17.6)		

NOTE. Values per 100.000 person-years.

* 2003–2004 and 2012–2013 incidence rates were age-standardized to the 1991–1992 population.

**Table 3:** Poisson regression model comparing major LEA rates by period, sex and age.

	Β	SE	P value	RR (95% CI)‡
Constant	-14.48	0.28	<0.001	
Period*				
1991–1992	-0.93	0.42	0.025	0.394 (0.175–0.888)
2003–2004	-0.87	0.41	0.033	0.420 (0.189–0.931)
Sex (male)	0.82	0.07	<0.001	2.276 (1.991–2.601)
Age†	0.08	0.01	<0.001	1.084 (1.076–1.092)
Age × 1991–1992	0.02	0.01	0.007	1.015 (1.004–1.026)
Age × 2003–2004	0.01	0.01	0.021	1.013 (1.002–1.023)

NOTE. Pearson *Χ*^2^ (113) = 131.4, p<0.001.

* Reference category is 2012–2013.

† Per year increase.

‡ Rate Ratio is calculated by e^Β^ (Wald 95% CI).

### Major LEA in the DM population

Crude IR of major LEA in the 2012–2013 DM population was 89.2 per 100.000 person-years (Table 4), which was a decrease compared to the 2003–2004 rate of 142.6 per 100.000 person-years (*χ*^2^(1) = 18.8, p<0.001). Among male DM patients crude IR decreased from 164.0 to 115.1 per 100.000 person-years (*χ*^2^(1) = 6.3, p = 0.012) and from 119.0 to 61.8 per 100.000 person-years (*χ*^2^(1) = 14.5, p<0.001) among female DM patients. Rate ratio of major LEA between DM patients in the DM population and non-DM patients in the general population in 2012–2013 was 18.8 (95%CI 15.2–23.2) for all ages and 8.4 (95%CI 6.9–10.5) in the population aged ≥45 years, with similar rates for men and women: 8.6 (95%CI 6.6–11.3) and 7.9 (95%CI 5.5–11.3) respectively (Table 4).

**Table 4:** Crude incidence rates of first ever major LEA for the DM population.

	Crude IR (95% CI)		P value*	RR (95% CI)†
	2003–2004	2012–2013		2012–2013
*Men and women*				
All ages	142.6 (120.7–167.3)	89.2 (76.9–102.8)	<0.001	18.8 (15.2–23.2)
Age ≥45	147.3 (124.6–173.0)	92.1 (79.3–106.4)	<0.001	8.4 (6.9–10.5)
*Men*				
All ages	164.0 (130.6–203.3)	115.1 (95.9–137.0)	0.012	19.6 (15.0–25.6)
Age ≥45	175.5 (139.6–217.8)	119.3 (99.1–142.4)	0.007	8.6 (6.6–11.3)
*Women*				
All ages	119.0 (92.2–151.1)	61.8 (47.6–78.9)	<0.001	16.9 (11.9–24.2)
Age ≥45	122.5 (94.9–155.6)	64.1 (49.3–82.1)	<0.001	7.9 (5.5–11.3)

NOTE. Values per 100.000 person-years.

* χ^2^ test of crude IR 2003–2004 vs 2012–2013, df = 1.

† Rate Ratio of crude IR for DM vs non-DM populations.

## Discussion

Comparing the incidence of major LEA in 2012–2013 with the 1991–1992 cohort, an increase was observed in terms of crude IR and absolute numbers of patients undergoing first ever major LEA. However, the age distribution in the Dutch population shifted considerably towards more elderly people[27]. Compared to 1991 there were 44% more people aged ≥45 years and 39% more people aged ≥75 years in 2013[23]. Considering that the prevalence of PAD and DM also increased in the 20 year time span[28,29], it is reassuring that compared to 1991–1992 and 2003–2004 decreased amputation rates were observed in 2012–2013 for the populations aged >63 and >70 years respectively. Following the 1989 St. Vincent Declaration which set up goals to improve the treatment of DM and reduce subsequent complications, considerable efforts were made in the Netherlands for the goals to be achieved in the next decade. The observed decreased incidence of major LEA among patients with DM, PAD or both diseases may be attributable to improvements of glycemic control[30], implementation of intensive multidisciplinary podotiatric care[12,15,31] and improved techniques in vascular surgery[32,33].

In terms of crude IR, age-standardized IR and RR, higher amputation rates were found among men compared to women, whereas women were older at the time of first major amputation in 2012–2013. These observations are similar to those in several other recent studies[8,34–36]. A clinical impression has been that dysvascular amputation patients are older at the time of first major LEA compared to previous two decades. However, in this study amputation patients in the most recent cohort were not older at the time of first major LEA compared to the previous cohorts. The decline of crude IR in the DM population is also reassuring, when we take into account that between 1999 and 2014 the prevalence of DM in the Netherlands had more than doubled[29], in which only 50% of this increase was attributable to the age distribution of the population. As expected, the crude IR in the DM populations were considerably higher compared to that of the general population. DM patients aged 0–40 years in our study were underrepresented in terms of absolute numbers compared to the all ages population. Therefore, we argue that we may only draw conclusions for patients aged ≥45, regarding the rate ratios of major LEA among DM versus non-DM populations.

Comparing incidence rates between regions is challenging due to differences in research methodology such as defining amputation levels, data being acquired at patient or aggregated levels and calculating rates for specific subpopulations (i.e., total, DM and non-DM populations)^3,4^. Therefore, our study focused on replicating previous studies in our region in order to more precisely compare the incidence rates and changes over time. To our knowledge, this is one of the few studies in which the data were extracted through patients’ records directly. To put the numbers in an international perspective, the decrease of major LEA incidence our study is similar to several recent studies[5,9,34,37]. Decreased major LEA incidence rates in the overall population were reported: from 23.3 to 16.0 per 100.000 person-years in Germany[5], from 23 to 12 per 100.000 person-years in the United States[37], from 14 to 9 per 100.000 person-years in Australia[38], whereas unchanged rates of 42.3 per 100.000 person-years were reported in Hungary[9]. Similar to our findings for the DM populations, recent studies reported decreased major LEA incidences: in Italy from 109 to 83 per 100.000 person-years[8], in Scotland from 187 to 111 per 100.000 person-years[39], in Finland from 94.4 to 48.3 per 100.000 person-years[36], whereas in Ireland[10] and the United Kingdom[35] unchanged incidence rates were reported, respectively 47 and 102 per 100.000 person-years. Although the definitions of level of amputation in the aforementioned studies are in line with ours, only two studies[36,39] accounted for multiple amputations per patient (i.e., bilateral and reamputation) in the same manner as we did. Also, except for the Finish researchers[36], none of the aforementioned studies applied our exclusion of patients with major LEA prior to the study periods. These factors may have contributed to systematically higher incidence rates in other studies. We argue that incidence rates should provide information about the number of patients being confronted with a major LEA for the first time. Because, when multiple amputations among the same patients are added up in IR calculation, any change of IR over time would be problematic to interpret. That is, readers may not be able to distinguish whether the rate of patients who have undergone amputation has changed, or the number of amputations being performed among a subset of high risk patients.

### Limitations

Data were collected only in the Northern three provinces of the Netherlands. The total population of this region was approximately 1.7 million people in 2013, which was 10.2% of the total Dutch population[23]. Although the distribution of age and sex in our region is similar to that of the Netherlands in general, we were unable to assess whether the health status was representative to the general population. Therefore, generalizability of the findings to the overall Dutch population may be somewhat limited. Our study design focused on major LEA as an outcome. In assessing amputation rates, it is important to distinguish total, minor and major LEA incidences[4]. Although some contradictory findings have been reported[4], several studies that report decreasing major LEA incidences also report increasing minor LEA rates[5,8,38]. A possible explanation is that more often than in the past, minor amputations are performed on patients at risk of limb amputation in order to prevent complication to major amputation. Lacking any data on minor LEA, we have no means of assessing whether our population followed the same trend.

The use of actual registry data for the denominators in the DM population is preferable to estimation using prevalence rates[35,39,40].Although recent research in the Netherlands has shown that estimated prevalence rates are similar to actual numbers[41], the incidence rates for the DM population in our study may be less precise due to relatively larger standard error for the younger age groups. Because the dataset for 1991–1992 did not contain separate variables for the presence of DM and PAD for each patient, we were unable to calculate IR for DM and non-DM populations for the 1991–1992 or take DM status into account in the main analysis of the IR change over time in the Poisson regression model. Crude incidence rates for the DM population and non-DM populations for the 2002–2004 and 2012–2013 cohorts are provided as supplemental data S3 Table. In line with a recent systematic review[4] we recommend that future research should preferably provide analysis of crude and age-standardized IR for the total, DM and non-DM populations.

### Clinical implication

Lower extremity amputations are associated with high mortality rates[42–44], have high costs in terms of socio-economic burden[43] and may cause considerable disability in the lives of patients confronted with the loss of a limb[45–47]. Though any amputation is an undesired outcome for patients, we argue that major LEA have worse clinical consequences: historically major LEA have been associated with lower functional status[48] and higher mortality rates compared to minor amputations[49]. Also, we should consider that patients undergoing minor LEA -including partial foot amputation- still do have a high risk of subsequent major LEA in their future[49]. Reduction of major LEA incidence on a regional level may therefore be regarded as a reflection of the effectiveness of health care services[50], with the reduction in the number of patients with PAD and/or DM undergoing a major LEA for the first time as the clinical outcome. However, a plateau of decreasing amputation rates may be reached in the future due to the relative increase in person-years of exposure to risk factors and the inevitability of amputation in some patients with advancing courses of PAD and DM.

## Conclusion

Age-standardized incidence rate of first ever dysvascular major LEA in 2012–2013 was 7.7 per 100.000 person-years. Amputation rates were more than twice as high among men compared to women. Although age distribution in the Dutch population shifted considerably toward more elderly people, major LEA rates for persons aged >63 years decreased in 2012–2013 compared to the 1991–1992 cohort. Crude incidence rate of major LEA for the DM population decreased from 142.6 per 100.000 person-years in 2003–2004 to 89.2 per 100.000 person-years in 2012–2013, in which the amputation rates for DM patients aged ≥45 years were more than 8 times higher compared to the non-DM population.

## Supporting information

S1 Table Details of search strategy and inclusion/exclusion criteria. (DOCX)

S2 Table Details and formulae illustrating calculations of age standardized incidence rates, confidence intervals and Poisson regression. (DOCX)

S3 Table Crude incidence rates for DM and non-DM populations. (DOCX)

S4 Table STROBE checklist. (DOC)

## Abbreviation

CI: confidence interval
DM: diabetes mellitus
IR: incidence rate
LEA: lower extremity amputation
PAD: peripheral arterial disease
RR: rate ratio
