# Supplementary material for: Incidence rates of dysvascular lower extremity amputation changes in Northern Netherlands: A comparison of three cohorts of 1991-1992, 2003-2004 and 2012-2013
Source: PLoS One. 2018 Sep 24;13(9):e0204623. doi: 10.1371/journal.pone.0204623 (PMC6152988; doi:10.1371/journal.pone.0204623)
Supplement: S1 Table — (DOCX) [file pone.0204623.s001.docx]

| **Inclusion** | **ICD-9 code(s)** |
| --- | --- |
| Amputation |  |
| Disarticulation of ankle | 84.13 |
| Disarticulation of ankle through malleoli of tibia and fibula | 84.13 |
| Other amputation below knee | 84.15 |
| Disarticulation of knee | 84.16 |
| Amputation above knee | 84.17 |
| Disarticulation of hip | 84.18 |
| Lower limb amputation, NOS | 84.10 |
| Peripheral arterial disease (PAD) |  |
| Atherosclerosis of the extremities,  unspecified | 440.20 |
| Atherosclerosis of the extremities with intermittent claudication | 440.21 |
| Atherosclerosis of the extremities with rest pain | 440.22 |
| Atherosclerosis of the extremities with ulceration | 440.23 |
| Atherosclerosis of the extremities with gangrene | 440.24 |
| Other peripheral vascular disease | 443.xx |
| Diabetes mellitus (DM)* | 250.xx |
|  |  |
| **Exclusion** |  |
| Malignant neoplasm of bone and articular cartilage | 170.xx |
| Malignant neoplasm of connective and other soft tissue | 171.xx |
| Malignant melanoma of skin | 172.xx |
| Traumatic amputation of leg(s) | 897.xx |
| Crushing injury of lower limb | 928.xx |
| Reflex sympathetic dystrophy of the lower limb | 337.22 |
| Certain congenital musculoskeletal deformities | 754.xx |
| Other congenital musculoskeletal anomalies | 756.xx |
| NOTE. First, extensive search terms were applied including ICD-9 codes, locally used operation/procedure codes and free text to identify all amputations within a time frame. Second, the exclusion criteria were applied. Third, sporadic cases clearly not related to PAD or DM (e.g. fulminant infection in otherwise healthy adults) were additionally excluded.  * For the 1991-1992 the variable was codified as dysvascular when patients had either PAD and/or DM, for the 2003-2004 and 2012-2013 cohorts separate variables were coded for PAD and DM. | |
