# Supplementary material for: Incidence rates of dysvascular lower extremity amputation changes in Northern Netherlands: A comparison of three cohorts of 1991-1992, 2003-2004 and 2012-2013
Source: PLoS One. 2018 Sep 24;13(9):e0204623. doi: 10.1371/journal.pone.0204623 (PMC6152988; doi:10.1371/journal.pone.0204623)
Supplement: S2 Table — (DOCX) [file pone.0204623.s002.docx]

| A. Age-standardized incidence rates (IR). Following the direct method for age standardization using the 1991-1992 as the reference population. Age was stratified in 5-year intervals in the study and reference subpopulations, e.g. 0-5, 5-10 years through 95 years and older. Age-standardized IR were obtained following:  $Age standardized IR= \sum_{i=1}^{20} (\text{ }a_{i}\text{ / }n_{i}\text{ })\times w_{i}$  In which:  a_i_ = N_subtotal amputees_  n_i_ = N_subtotal actual population_  w_i_ = proportion_subtotal reference population_ |
| --- |
| B. Confidence intervals for age-standardized IR expressed as:  Age-standardized IR ± Z_α/2_ × SE(Age-standardized IR)  SE (Age-standardized IR) = √Var(Age-standardized IR)  $\mathrm{Var}\left( Age standardized IR \right)= \frac{\sum_{i=1}^{20} ( a_{i}\text{×}{w_{i}}^{2}\times100000 ) / n_{i}}{\sum_{i=1}^{20} ( {w_{i})}^{2}}$  In which:  SE = standard error  Var = variance  a_i_ = age specific rate per 5-years age category w_i_ = person-years in the reference population per 5-years age category n_i_ = person-years in the actual population per 5-years age category |
| C. Poisson regression equation for obtaining rate ratio (RR) for an interaction effect.  $Ln\left( \lambda\right)=\beta_{1}X_{1}+ \beta_{2}X_{2}+\ldots\beta_{k}X_{k}$  As applied in this article, for example for age 75 years for RR of period 2012-2013 vs 1991-1992:  $Ln\left( \lambda\right)=-0.93+ 0.015 \times75=0.178$ $e^{0.178}=1.195$ $RR=\frac{1}{1.195}=0.837$ |
| D. Estimation of DM population for crude incidence rate (IR).  $N\_DM= \sum_{i=1}^{5} \left( \text{ }n_{0-15years}\text{ }\times\text{ }{prev}_{0-15years} \right)+\left( \text{ }n_{i}\text{ }\times\text{ }{prev}_{i} \right)$  In which: N_DM = N_total DM population_  n_i_ = N_subpopulation by 15-years age groups_  prev_i_ = prevalence rate_by 15-years age groups_ |
|  |
