# Supplementary material for: Incidence rates of dysvascular lower extremity amputation changes in Northern Netherlands: A comparison of three cohorts of 1991-1992, 2003-2004 and 2012-2013
Source: PLoS One. 2018 Sep 24;13(9):e0204623. doi: 10.1371/journal.pone.0204623 (PMC6152988; doi:10.1371/journal.pone.0204623)
Supplement: S3 Table — (DOCX) [file pone.0204623.s003.docx]

|  | **DM population crude IR (95% CI)** | |  | **Non-DM population crude IR (95% CI)** | |  | **RR (95% CI)†** | |
| --- | --- | --- | --- | --- | --- | --- | --- | --- |
|  | **2003-2004** | **2012-2013** |  | **2003-2004** | **2012-2013** |  | **2003-2004** | **2012-2013** |
| *Men and women* |  |  |  |  |  |  |  |  |
| All ages | 142.6 (120.7-167.3) | 89.2 (76.9-102.8) |  | 4.5 (3.8-5.3) | 4.8 (4.0-5.5) |  | 31.5 (25.1-39.5) | 18.8 (15.2-23.2) |
| Age ≥45 | 147.3 (124.6-173.0) | 92.1 (79.3-106.4) |  | 11.6 (9.8-13.6) | 10.8 (9.2-12.7) |  | 12.9 (10.3-16.3) | 8.4 (6.9-10.5) |
| *Men* |  |  |  |  |  |  |  |  |
| All ages | 164.0 (130.6-203.3) | 115.1 (95.9-137.0) |  | 5.8 (4.8-7.0) | 5.9 (4.8-7.1) |  | 28.1 (20.9-37.7) | 19.6 (15.0-25.6) |
| Age ≥45 | 175.5 (139.6-217.8) | 119.3 (99.1-142.4) |  | 15.3 (12.5-18.7) | 13.8 (11.2 16.8) |  | 11.5 (8.5- 15.4) | 8.6 (6.6-11.3) |
| *Women* |  |  |  |  |  |  |  |  |
| All ages | 119.0 (92.2-151.1) | 61.8 (47.6-78.9) |  | 3.3 (2.5-4.2) | 3.6 (2.8-4.7) |  | 36.4 (25.4- 52.0) | 16.9 (11.9-24.2) |
| Age ≥45 | 122.5 (94.9-155.6) | 64.1 (49.3-82.1) |  | 7.7 (5.8-10.0) | 8.1 (6.2-10.4) |  | 15.9 (11.0- 22.8) | 7.9 (5.5-11.3) |
| NOTE. Values per 100.000 person-years. | | | | | | | | |
